# Supplementary material for: Association between oral microbiome and seven types of cancers in East Asian population: a two-sample Mendelian randomization analysis
Source: Front Mol Biosci. 2023 Nov 21;10:1327893. doi: 10.3389/fmolb.2023.1327893 (PMC10702768; doi:10.3389/fmolb.2023.1327893)
Supplement: Supplementary file 1 [file DataSheet1.ZIP › Supplementary Materials/MR plots for tongue/tongue═╝/Lung cancer/pheno.2004_to_lung cancer_leave_one_out.pdf]

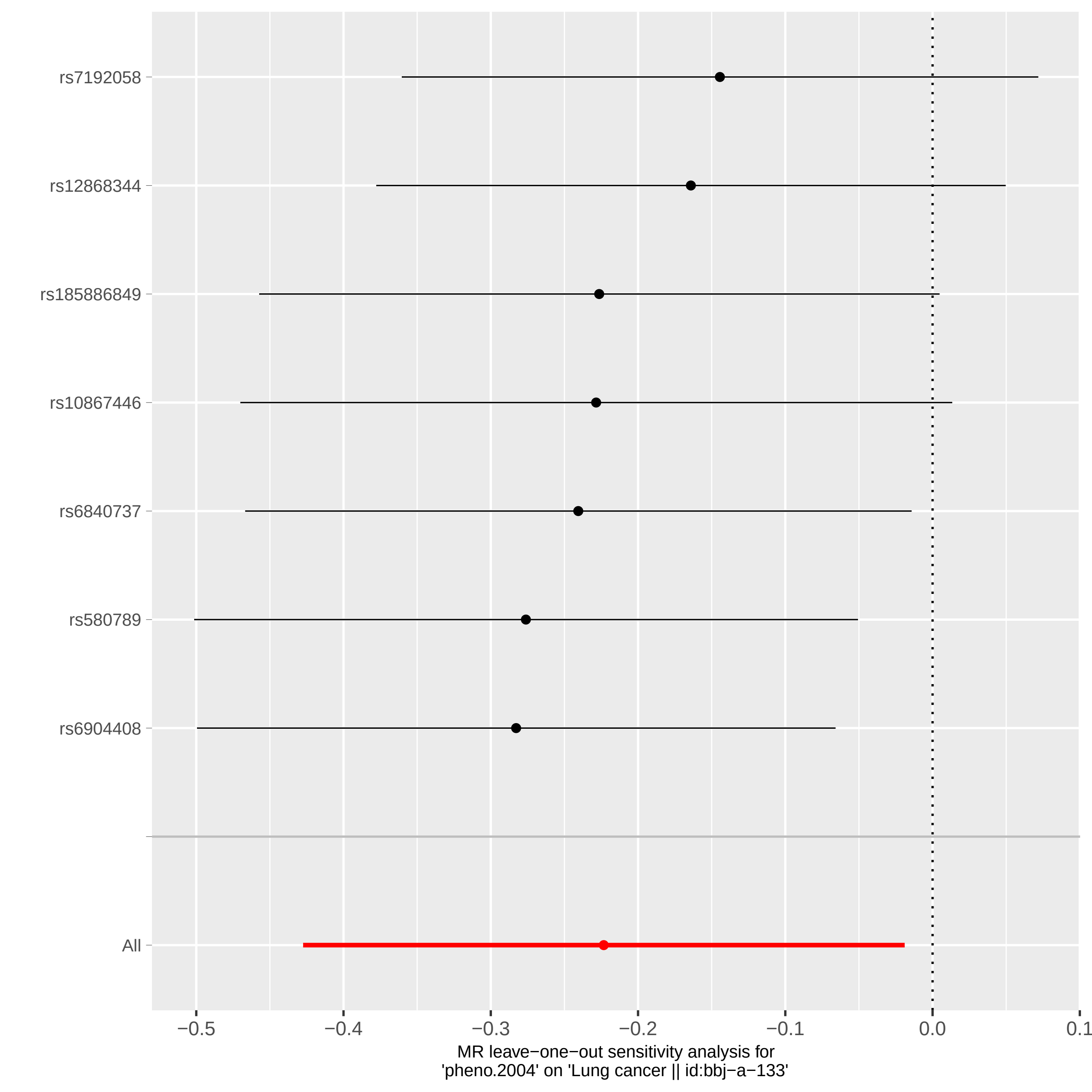
rs7192058
rs12868344
rs185886849
rs10867446
rs6840737
rs580789
rs6904408
All
−0.5
−0.4
−0.3
−0.2
−0.1
0.0
0.1
MR leave−one−out sensitivity analysis for
'pheno.2004' on 'Lung cancer || id:bbj−a−133'
